# Telehealth Usage in Otolaryngology: A Comparative Study Pre‐ and Post‐COVID‐19

**DOI:** 10.1002/oto2.109

**Published:** 2024-01-27

**Authors:** Max L. Lee, Eric X. Wei, Cherian Kandathil, Sam P. Most

**Affiliations:** ^1^ Department of Otolaryngology–Head and Neck Surgery Stanford Health Care Palo Alto California USA

**Keywords:** access, COVID‐19, demographics, health equity, telehealth, telemedicine

## Abstract

The COVID‐19 pandemic led to increased telehealth utilization in outpatient otolaryngology settings. While other studies on telehealth usage in otolaryngology settings have focused on demographic disparities during the pandemic, none have yet assessed how these demographic disparities have evolved from before versus after the pandemic. This study examines 4 recent consecutive years of demographic and clinical data from a large hospital system to investigate how the COVID‐19 pandemic has changed demographic patterns in telehealth utilization. We demonstrate substantial increases in the number of otolaryngology patients participating in telehealth since the beginning of the COVID‐19 pandemic but with no differences in patient distributions by race or ethnicity over time. We also found that telehealth patients, on average, were younger, more likely to be English‐speaking, and more likely to be female. While these disparities widened slightly after the start of the pandemic, they were also present prior to the pandemic.

The early COVID‐19 pandemic led to surgical delays and increased telehealth usage in outpatient otolaryngology settings.^1^, ^2^ Prepandemic, the telehealth literature was focused on its application in rural and underserved areas.^3^, ^4^, ^5^, ^6^ Since the pandemic, telehealth infrastructure has expanded substantially.^3^ Despite the growing popularity and utility of telehealth, there are concerns that increased telehealth usage may exacerbate health disparities.

A growing body of literature has described disparities in telehealth usage by age, language, race/ethnicity, and income during the pandemic.^6^, ^7^, ^8^, ^9^, ^10^ One study of an academic tertiary care practice found that older, male, and lower‐income patients were less likely to complete an otolaryngology telehealth visit, but did not find differences by race/ethnicity.^6^ A larger and broader study found that older, non‐English speaking, and Asian patients had lower telemedicine utilization.^7^ While these studies characterized disparities in telehealth usage during the pandemic, none have described how demographic differences in otolaryngology telehealth usage have evolved since before the pandemic. Our aim is to investigate how the pandemic contributed to demographic changes in otolaryngology telehealth by examining 4 recent consecutive years of data (2019‐2022).

## Methods

We obtained nonidentified clinical data using the STAnford Research Repository (STARR), which collects patient demographic and clinical information from patients treated at Stanford‐affiliated clinics. This study was approved by the Stanford Institutional Review Board (IRB #64700). Adult otolaryngology patients seen via telehealth at 3 Stanford‐affiliated clinics were included in this study, between January 1, 2019 and December 31, 2022. These clinics included the main university hospital, one comprehensive otolaryngology clinic, and one otology/neurotology clinic. Telehealth visits were defined as either telephone calls or virtual outpatient visits. We obtained patient demographic characteristics including age, race/ethnicity, gender, and primary language. We utilized *t*‐tests and chi‐square analyses to compare demographic characteristics of telehealth patients in 2019 versus 2020 to 2022. For all analyses, a *P* < .05 was considered significant. All analyses were completed using Stata 14 (Stata Corp.).

## Results

**Figure 1:**
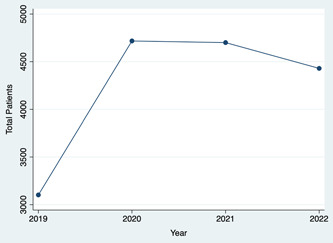
Total number of telehealth patients annually, 2019 to 2022.

The average age of otolaryngology telehealth patients decreased between 2019 and 2020 to 2022 (57.52 years in 2019 vs 55.32 years in 2020‐2022, *P* < .0001; Table 1). Additionally, the number of telehealth patients increased from 3102 in 2019 to an average of 4616 telehealth patients annually from 2020 to 2022 (Figure 1). The percentage of white, non‐Hispanic patients as a proportion of total patients decreased from 53.38% in 2019 to 51.55% in 2020 to 2022, driven primarily by an increase in the proportion of patients classified as other or unknown from 24.76% to 27.03% (*P* = .112). The percentage of female patients as a proportion of total patients increased by 3.17% from 53.24% in 2019 to 56.41% in 2020 to 2022 (*P* = .006), while the proportion of English‐speaking patients increased by 2.02% from 89.72% in 2019 to 91.74% in 2020 to 2022.

**Table 1:** Descriptive Statistics for Demographic Characteristics, 2019 versus 2020 to 2022

	Year		
	*2019*	*2020‐22*	
Observations	3102	13848	Chi‐square *P* value
Age (in years)			
Mean	57.516	55.319	
Std. Error	0.329	0.157	
95% CI	56.872‐58.160	55.012‐55.626	
*P* value	***P*** < **.0001**		
Race/Ethnicity			*P* = .112
Hispanic/Latino	94 (3.03)	414 (2.99)	
White, non‐Hispanic	1656 (53.38)	7139 (51.55)	
Black, non‐Hispanic	95 (3.06)	380 (2.74)	
Asian	489 (15.76)	2173 (15.69)	
Other/Unknown	768 (24.76)	3743 (27.03)	
Gender			***P*** = **.006**
Female	1652 (53.24)	7812 (56.41)	
Male	1450 (46.73)	6037 (43.59)	
Language			***P*** < **.001**
English	2783 (89.72)	12,705 (91.74)	
Other	319 (10.28)	1144 (8.26)	

*Note*: Statistically significant *P* values are shown in bold font.

**Figure 2:**
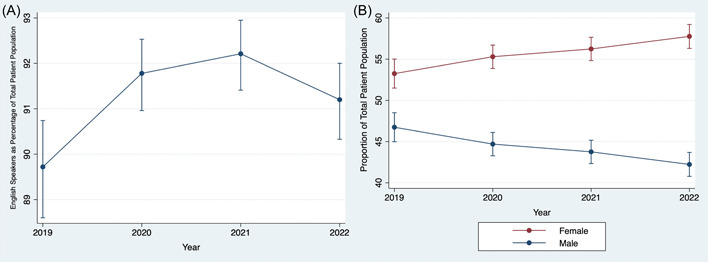
Changes in demographic characteristics among telehealth participants, 2019 to 2022. (A) English speakers as a proportion of total patient population. (B) Gender distribution of patient population.

When looking at annual trends in percentage of English‐speaking patients, the largest 1‐year change was between 2019 and 2020 (2.06 percentage points), with smaller annual changes occurring between 2020 and 2022. The proportion of English‐speaking patients decreased in 2022 relative to 2020 and 2021, with overlapping 95% confidence intervals when comparing 2022 to 2019 (Figure 2A). Gender showed a steadier and more persistent annual change from 2019 to 2022, with an average increase of 1.50% in proportion of female patients each year (Figure 2B).

## Discussion

Our study demonstrates substantial increases in the number of otolaryngology patients participating in telehealth postpandemic. Additionally, we demonstrate that postpandemic, telehealth patients were younger and more likely to be English‐speaking and female. These disparities were present prepandemic and either widened or persisted through the pandemic. Low rates of telehealth adoption among non‐English speakers have also been documented in other studies, with potential explanations including challenges with real‐time translation and with navigating telehealth software.^9^, ^11^

We found that the proportion of female otolaryngology telehealth patients increased annually, widening the gender gap that was documented prepandemic.^7^, ^9^, ^12^ One potential explanation is higher levels of eHealth literacy among women,^13^ which could have led to quicker uptake of telehealth during the pandemic. Additionally, caregiving responsibilities intensified during the pandemic, and women, who disproportionately assume family caregiver roles, may have preferred the convenience of telehealth to a greater degree than men,^14^ although future work is needed to validate these hypotheses.

We did not find substantial differences in patient distributions by race/ethnicity over time. Several studies comparing telehealth and in‐person visits during the pandemic found mixed results, with some reporting no differences and others finding that racial/ethnic minorities were less likely to complete telehealth visits.^15^, ^16^, ^17^ However, since many of these studies were limited to individual hospital systems,^6^, ^15^, ^16^ these findings, when taken together, likely suggest that disparities in telehealth usage may vary significantly by region.

This study has several limitations. Our patient population lives in a region of the country that is a major technology center, and health technologies including telemedicine were adopted at a faster rate prepandemic than most other parts of the country. Additionally, since the STARR database only documents first preferential race, multiracial patients are not accurately represented. Despite these limitations, our study is the first to document how demographic patterns in otolaryngology telehealth usage have evolved from prepandemic to postpandemic. Interesting future research could determine whether telehealth disparities are associated with treatment delays or worsened outcomes for important otolaryngology diagnoses, such as tumors or pediatric hearing loss. While solutions to improve accessibility for older and non‐English speaking patients have been proposed, such as technology literacy outreach programs or mobile translation apps, future work should investigate ways to improve upon these solutions.^18^, ^19^

## Author Contributions

**Max L. Lee**, design, data analysis, writing of original draft, review, and editing; **Eric X. Wei**, design, writing of original draft, review, and editing; **Cherian Kandathil**, design, review and editing; **Sam P. Most**, review and editing.

## Disclosures

### Competing interests

None.

### Funding source

None.
